# Tracing the Distribution of European Lactase Persistence Genotypes Along the Americas

**DOI:** 10.3389/fgene.2021.671079

**Published:** 2021-09-22

**Authors:** Ana Cecília Guimarães Alves, Natalie Mary Sukow, Gabriel Adelman Cipolla, Marla Mendes, Thiago P. Leal, Maria Luiza Petzl-Erler, Ricardo Lehtonen Rodrigues Souza, Ilíada Rainha de Souza, Cesar Sanchez, Meddly Santolalla, Douglas Loesch, Michael Dean, Moara Machado, Jee-Young Moon, Robert Kaplan, Kari E. North, Scott Weiss, Mauricio L. Barreto, M. Fernanda Lima-Costa, Heinner Guio, Omar Cáceres, Carlos Padilla, Eduardo Tarazona-Santos, Ignacio F. Mata, Elena Dieguez, Víctor Raggio, Andres Lescano, Vitor Tumas, Vanderci Borges, Henrique B. Ferraz, Carlos R. Rieder, Artur Schumacher-Schuh, Bruno L. Santos-Lobato, Pedro Chana-Cuevas, William Fernandez, Gonzalo Arboleda, Humberto Arboleda, Carlos E. Arboleda-Bustos, Timothy D. O’Connor, Marcia Holsbach Beltrame, Victor Borda

**Affiliations:** ^1^Laboratório de Genética Molecular Humana, Departamento de Genética, Setor de Ciências Biológicas, Universidade Federal do Paraná, Curitiba, Brazil; ^2^Programa de Pós-Graduação em Genética, Departamento de Genética, Setor de Ciências Biológicas, Universidade Federal do Paraná, Curitiba, Brazil; ^3^Laboratório de Diversidade Genética Humana, Departamento de Genética, Ecologia e Evolução, Instituto de Ciências Biológicas, Universidade Federal de Minas Gerais, Belo Horizonte, Brazil; ^4^Laboratório de Polimorfismos e Ligação, Departamento de Genética, Setor de Ciências Biológicas, Universidade Federal do Paraná, Curitiba, Brazil; ^5^Laboratório de Polimorfismos Genéticos, Departamento de Biologia Celular, Embriologia e Genética, Centro de Ciências Biológicas, Universidade Federal de Santa Catarina, Florianópolis, Brazil; ^6^Laboratorio de Biotecnología y Biología Molecular, Instituto Nacional de Salud, Lima, Peru; ^7^Emerging Diseases and Climate Change Research Unit, School of Public Health and Administration, Universidad Peruana Cayetano Heredia, Lima, Peru; ^8^Institute for Genome Sciences, University of Maryland School of Medicine, Baltimore, MD, United States; ^9^Division of Cancer Epidemiology and Genetics, National Cancer Institute (NCI), National Institutes of Health (NIH), Bethesda, MD, United States; ^10^Department of Epidemiology and Population Health, Albert Einstein College of Medicine, Bronx, NY, United States; ^11^Division of Public Health Sciences, Fred Hutchinson Cancer Research Center, Seattle, WA, United States; ^12^Department of Epidemiology, Gillings School of Global Public Health, The University of North Carolina at Chapel Hill, Chapel Hill, NC, United States; ^13^Channing Division of Network Medicine, Department of Medicine, Brigham and Women’s Hospital and Harvard Medical School, Boston, MA, United States; ^14^Universidade Federal da Bahia, Instituto de Saúde Coletiva, Salvador, Brazil; ^15^Fundação Oswaldo Cruz, Centro de Integração de Dados e Conhecimentos para Saúde (Cidacs), Salvador, Brazil; ^16^Fundação Oswaldo Cruz, Instituto René Rachou, Belo Horizonte, Brazil; ^17^Universidade Federal de Minas Gerais, Programa de Pós-Graduação em Saúde Pública, Belo Horizonte, Brazil; ^18^Facultad de Ciencias de la Salud, Universidad de Huánuco, Huánuco, Peru; ^19^Carrera de Medicina Humana, Facultad de Ciencias de la Salud, Universidad Científica del Sur, Lima, Peru; ^20^Veterans Affairs Puget Sound Health Care System, Seattle, WA, United States; ^21^Department of Neurology, University of Washington, Seattle, WA, United States; ^22^Lerner Research Institute, Genomic Medicine, Cleveland Clinic, Cleveland, OH, United States; ^23^Neurology Institute, Universidad de la República, Montevideo, Uruguay; ^24^Department of Genetics, Facultad de Medicina, Universidad de la República, Montevideo, Uruguay; ^25^Ribeirão Preto Medical School, Universidade de São Paulo, Ribeirão Preto, Brazil; ^26^Movement Disorders Unit, Department of Neurology and Neurosurgery, Universidade Federal de São Paulo, São Paulo, Brazil; ^27^Departamento de Neurologia, Universidade Federal de Ciências da Saúde de Porto Alegre, Porto Alegre, Brazil; ^28^Serviço de Neurologia, Hospital de Clínicas de Porto Alegre, Porto Alegre, Brazil; ^29^Departamento de Farmacologia, Universidade Federal do Rio Grande do Sul, Porto Alegre, Brazil; ^30^Instituto de Ciências da Saúde, Universidade Federal do Pará, Belém, Brazil; ^31^CETRAM, Facultad de Ciencias Médicas, Universidad de Santiago de Chile, Santiago, Chile; ^32^Neuroscience and Cell Death Research Groups, Medical School and Genetic Institute, Universidad Nacional de Colombia, Bogotá, Colombia; ^33^Program for Personalized and Genomic Medicine, School of Medicine, University of Maryland, Baltimore, Baltimore, MD, United States; ^34^Department of Medicine, School of Medicine, University of Maryland, Baltimore, Baltimore, MD, United States

**Keywords:** *–13910C* > *T*, *MCM6* gene, lactose intolerance, dairy consumption, nutrition policies, Latin America, population genetics

## Abstract

In adulthood, the ability to digest lactose, the main sugar present in milk of mammals, is a phenotype (lactase persistence) observed in historically herder populations, mainly Northern Europeans, Eastern Africans, and Middle Eastern nomads. As the *–13910^∗^T* allele in the *MCM6* gene is the most well-characterized allele responsible for the lactase persistence phenotype, the *–13910C* > *T* (rs4988235) polymorphism is commonly evaluated in lactase persistence studies. Lactase non-persistent adults may develop symptoms of lactose intolerance when consuming dairy products. In the Americas, there is no evidence of the consumption of these products until the arrival of Europeans. However, several American countries’ dietary guidelines recommend consuming dairy for adequate human nutrition and health promotion. Considering the extensive use of dairy and the complex ancestry of Pan-American admixed populations, we studied the distribution of *–13910C* > *T* lactase persistence genotypes and its flanking haplotypes of European origin in 7,428 individuals from several Pan-American admixed populations. We found that the *–13910^∗^T* allele frequency in Pan-American admixed populations is directly correlated with allele frequency of the European sources. Moreover, we did not observe any overrepresentation of European haplotypes in the *–13910C* > *T* flanking region, suggesting no selective pressure after admixture in the Americas. Finally, considering the dominant effect of the *–13910^∗^T* allele, our results indicate that Pan-American admixed populations are likely to have higher frequency of lactose intolerance, suggesting that general dietary guidelines deserve further evaluation across the continent.

## Introduction

Lactase phlorizin hydrolase, popularly known as lactase, is an enzyme expressed by enterocytes from the small intestine. Lactase is responsible for the hydrolysis of lactose, a non-absorbable sugar present in milk of mammals, into glucose and galactose, which in turn are simple sugars absorbable by the intestinal mucosa (Mantei et al., 1988). In mammals, lactase is highly expressed by newborns; after weaning, however, its expression is naturally downregulated (Wang et al., 1994), leading to a phenotype known as lactase non-persistence (LNP). This trait, also known as adult-type hypolactasia, is related to lactose indigestion and malabsorption. It may progress to lactose intolerance associated with indigestion, bloating, abdominal pain, nausea, vomiting, flatulence, and diarrhea (Auricchio et al., 1963; Boll et al., 1991). In contrast, a high prevalence of lactase persistence (LP) occurs in populations with a long history of pastoralism and dairy consumption, mainly in Northern Europe and among nomads in Africa and the Middle East (Ingram et al., 2009). Single-nucleotide polymorphisms (SNPs) in the *MCM6* gene, located −14 kb upstream of the *LCT* gene, in its enhancer region, are believed to be responsible for the LP phenotype. The *–13910^∗^T* allele (*–13910C* > *T*, rs4988235, intron 13, *MCM6* gene) is widely distributed in the European population; the allele frequencies vary from 8.9% in Tuscany (Italy) to 72.0% among British (England and Scotland), with an average of 50.8% in Europe (1000 Genomes Project Consortium, Auton et al., 2015). The *T* allele enhances *LCT* expression at the mRNA level, causing LP in adulthood, even among heterozygotes, thus determining a dominant trait (*TT* and *TC* genotypes) (Enattah et al., 2002). In Asia, despite the occurrence of pastoralist populations, the LP phenotype frequency is lower than that reported in Europe and Africa (Ségurel and Bon, 2017), and the *–13910^∗^T* allele frequencies rarely exceed 10% (Itan et al., 2010). Other SNPs in African and Middle Eastern populations are observed in the same *MCM6* gene region (intron 13) and are also associated with the LP phenotype. Among these, the most well-known are *–14010G* > *C* (rs145946881), *–14009T* > *G* (rs869051967), *–13915T* > *G* (rs41380347), and *–13907C* > *G* (rs41525747) (Tishkoff et al., 2007; Ranciaro et al., 2014; Liebert et al., 2017).

In the Americas, archaeozoological evidence supports the occurrence of mammal domestication around 7,000–6,000 years ago and included the ancestors of modern llamas, alpacas, vicuñas, and guanacos (Wheeler, 1995; Kadwell et al., 2001). Despite breeding these animals, there is no archeological or cultural evidence of dairy consumption by Native American populations until Europeans arrived in the late fifteenth century (Gade, 1999) – cattle were not introduced in the Americas until 1493 (Primo, 1992). Not surprisingly, studies regarding living Native Americans from Brazil (Friedrich et al., 2012a), Chile (Fernández et al., 2016), Ecuador (Paz-Y-Miño et al., 2016), Mexico (Ojeda-Granados et al., 2016), Peru (Figueroa et al., 1971), and the United States (Duncan and Scott, 1972; Casey, 2005) identified lower LP phenotype frequencies in these populations (20% on average), suggesting that most people cannot digest lactose naturally. Countries such as Peru, Mexico, and Chile have a high proportion of Native American ancestry – 80, 57.5, and 49.3%, respectively (Ruiz-Linares et al., 2014; Adhikari et al., 2016; Harris et al., 2018) – which could explain the low frequency of the LP phenotype. Although there are no *–13910^∗^T* allele frequencies reported in ancient Native American samples, the low frequencies observed in Asian peoples and present-day Native Americans suggest that the *–13910^∗^T* allele was introduced in the Americas after the arrival of Europeans. Moreover, African and Middle Eastern SNPs have been reported at very low frequencies in the Americas. The frequency of *–14010^∗^C* (*–14010G* > *C*, rs145946881) in Afro-Brazilians from the South is 0.27% (Friedrich et al., 2012b); the frequencies of *–14011^∗^T* (*–14011C* > *T*, rs4988233) in admixed Brazilians from the North and the Northeast are 0.25 and 0.58%, respectively (Friedrich et al., 2012b); and the frequencies of *–13913^∗^C* (*–13913T* > *C*, rs4145614) and *–13915^∗^G* (*–13915T* > *G*, rs41380347) in Mestizos from Ecuador are 0.20 and 0.50%, respectively (Paz-Y-Miño et al., 2016). Therefore, the phenotypic variation observed is mainly due to the *–13910C* > *T* (rs4988235) substitution of European origin. Thus, the admixture events that led to the formation of current Latin American populations contributed to introducing LP-associated alleles in these groups (Friedrich et al., 2012a; Mendoza Torres et al., 2012; Latorre et al., 2014; Fernández et al., 2016; Ojeda-Granados et al., 2016; Paz-Y-Miño et al., 2016; Valencia et al., 2017; Montalva et al., 2019). Consequently, higher frequencies of the LP phenotype are observed in admixed populations from Brazil (Friedrich et al., 2012b), and Mestizos from Chile (Fernández et al., 2016), Colombia (Mendoza Torres et al., 2012), Ecuador (Paz-Y-Miño et al., 2016), and Mexico (Ojeda-Granados et al., 2016).

Because of the Latin American population formation process, genetic analyses have revealed the heterogeneous pattern of ancestries across the continent (Kehdy et al., 2015; Chacón-Duque et al., 2018; Harris et al., 2018; Soares-Souza et al., 2018). The peopling of the Americas occurred about 20,000 years ago, when an East Asian–derived group accessed the continent through the Bering Strait (Goebel et al., 2008; Dillehay, 2009; Reich et al., 2012; Mendes et al., 2020). Sequential population divisions and little gene flow from other continents after divergence gave rise to distinct Native American populations, with highly differentiated population groups, such as the Mesoamericans, Andeans, and Amazonians, distributed across the continent (Greenberg et al., 1986; Salzano, 2011; Reich et al., 2012; Mendes et al., 2020). Later on, during the Colonial period, Europeans brought about 9 million Africans through the Transatlantic Slave Trade to the Americas (Wehling et al., 1994; John, 1997; de Mello e Souza, 2007). Although there are no official historical records of the subcontinental origin of African peoples, previous genetic studies confirmed that West-Central African ancestry is the most prevalent in the Americas (Gouveia et al., 2020). Further on, between the nineteenth and twentieth centuries, European immigration was intensified mostly in South America. The resulting admixture drastically modified the genetic makeup of the continent.

The complex demographic processes involved in the formation of the Americas gave rise to mosaic populations in which ancestry proportions vary among and within the countries, affecting the distribution of Mendelian and complex phenotypes (Pena et al., 2011, 2020; Ruiz-Linares et al., 2014; Adhikari et al., 2016). Considering this, in the present study, we (i) addressed the geographical distribution of the *–13910C* > *T* SNP across the Americas, as well as (ii) analyzed this locus for evidence of positive selection in Pan-American admixed populations (American continent populations, i.e., North, Central, and South Americas). Moreover, we discussed our results in light of the use of dairy products in public health policies for nourishment in Latin American countries.

## Materials and Methods

### Publicly Available Datasets

We analyzed genomic information of 7,428 unrelated individuals from North, Central, and South Americas (Supplementary Table 1). This includes whole-genome sequencing (WGS) data from admixed individuals of the 1000 Genomes Project (1000 Genomes Project Consortium, Auton et al., 2015) [African Americans from Southwest United States (ASW); African Caribbeans from Barbados (ACB); individuals of Mexican ancestry from Los Angeles, United States (MXL); Puerto Ricans from Puerto Rico (PUR); and Colombians from Medellin (CLM)], as well as WGS data from individuals of the TOPMED Project (Taliun et al., 2021) (individuals from the Dominican Republic and Cuba from HCHS/SOL cohort, and individuals from Costa Rica from CRA cohort) and the Peruvian Genome Project (Harris et al., 2018). We also included genotype array data from the LARGE-PD Project (Loesch et al., 2020) (individuals from Brazil, Colombia, Chile, and Uruguay), two Brazilian EPIGEN population-based cohorts (Kehdy et al., 2015) (individuals from Salvador and Bambui), and from admixed and Native American individuals of the Peruvian Genome Project (Harris et al., 2018; Borda et al., 2020). All these datasets were generated from different sources (Supplementary Table 1) but include the *–13910C* > *T* SNP (rs4988235). In order to keep a higher SNP density for haplotype-based analyses, we organized the genomic information in two datasets: (i) LARGE-PD only and (ii) a merged dataset (including all other datasets). During the quality control of these two datasets, we excluded SNPs with significant missing data (>10%), loci with 100% of heterozygosity, non-chromosomal information, A/T–C/G genotypes, and SNPs with minor allele count = 1 using PLINK 1.9 (Chang et al., 2015). For the merging process, we used the flags –bmerge and –flip when necessary. Finally, we kept biallelic SNPs and removed singletons and monomorphic positions as they are not informative for population genetic analyses. We ended with 1,010,078 and 1,528,206 SNPs for the LARGE-PD Project and the merged dataset, respectively.

We also considered allele frequency information of ancient Native Americans from Posth et al. (2018) and Nakatsuka et al. (2020) and of European populations [Utah residents (CEPH) with Northern and Western European ancestry (CEU), British from England and Scotland (GBR), Finnish from Finland (FIN), Iberian from Spain (IBS), and individuals from Toscani in Italy (TSI)] from the 1000 Genomes Project (Supplementary Table 1).

### Newly Generated Datasets

Moreover, we generated sequencing data for the *LCT* enhancer (*MCM6* gene, intron 13) for 259 Afro-Brazilians individuals from the south region of Brazil. The sample included 241 individuals from Curitiba and its metropolitan region (Paraná state) and 18 individuals from the quilombola community of Sertão do Valongo (Santa Catarina state) (Supplementary Figure 1). For Curitiba individuals, first-degree relatives were excluded based on self-declared information. Blood samples had been previously collected as authorized under the Brazilian CONEP (Comissão Nacional de Ética em Pesquisa) registry numbers 180/2001 and 2.970.200 (CAAE: 02727412.4.0000.0096). These participants were classified as Afro-Brazilians. For the quilombola community, blood samples were collected according to the ethical guidelines in effect at that time, and individuals were classified as Afro-Brazilians considering the settlement history and isolation of the community described elsewhere (Souza and Culpi, 1992, 2005). All participants gave their written informed consent. DNA was extracted either by the phenol–chloroform–isoamyl alcohol method (Sambrook et al., 1989) or by the salting-out method (Lahiri and Nurnberger, 1991).

### Sequencing

The Afro-Brazilians sample (*n* = 259) was sequenced for a fragment of 594 bp in the *MCM6* gene, including the *–13910C* > *T* SNP (rs4988235). The fragment was amplified by polymerase chain reaction (PCR) on a Mastercycler EP Gradient S^®^ (Eppendorf, Germany). The forward and reverse primers used were as follows: 5’-GGCAGGGGTTTGGAACTTTC-3’ and 5’-CTGTTGAATGCTCATACGACCA-3’, respectively. Other reagents and the protocol used are described in Supplementary Tables 2, 3, respectively.

The PCR products were purified using exonuclease I (Fermentas, United States) and alkaline phosphatase (Thermo Fisher Scientific, United States) on a Mastercycler EP Gradient S^®^ (Eppendorf) at 37°C for 1 h and 80°C for 15 min. The sequencing was performed using the same primers used in the PCR and BigDye^®^ Terminator Cycle Sequencing Standard v3.1 (Life Technologies, United States), according to the instructions of the manufacturer. Sequencing reactions consisted of a first step at 95°C for 1 min, followed by 25 cycles of 95°C for 10 s, 50°C for 5 s, and 60°C for 4 min. The sequencing products were purified using ethanol (Merck, Germany), resuspended in Hi-Di Formamide (Life Technologies), and, finally, submitted to capillary electrophoresis in a 3500xl Genetic Analyzer Sequencer (Life Technologies).

We analyzed the obtained sequences using the Mutation Surveyor^®^ 3.30 software (SoftGenetics, United States), which aligns the forward and reverse sequences to a human genome reference sequence (GRCh38) available in NCBI (National Center for Biotechnology Information) resources, enabling the evaluation of amplified sequences and the genotype annotation for the *–13910C* > *T* SNP (Supplementary Table 4). The sequences were deposited in the GenBank at NCBI website under the accession numbers MZ362598 to MZ362856.

### Allelic and Genotypic Frequencies for the *–13910^∗^T* Variant

We calculated allelic frequencies for the *–13910^∗^T* allele (rs4988235) in each population using the –extract and –freq flags of PLINK 1.9. For genotype frequencies, we used the –hardy flag, which also calculates whether the population is in Hardy–Weinberg equilibrium for the given locus.

### Ancestry Analyses

Global and local ancestry inferences were performed for each dataset. For both analyses, we merged each dataset with a reference panel of 848 individuals from four continental ancestries: 206 European (IBS and CEU), 207 African (LWK and YRI), and 207 East Asian (CDX and JPT) individuals from the 1000 Genomes Project, and 228 unadmixed Native American individuals from the Peruvian Genome Project (Borda et al., 2020). As the genetic information available for Afro-Brazilians from Curitiba and Sertão do Valongo corresponds only to the *MCM6* region, it was not possible to perform global or local ancestry analyses for these samples. Instead, we extracted the global ancestry information for Sertão do Valongo individuals from Luizon (2007), who performed this inference using eight ancestry-informative markers in the same population. No data that could enable this analysis were available for Afro-Brazilians from Curitiba.

For global ancestry analysis, we removed linked variants (*r*^2^ > 0.1) using the PLINK flag –indep-pairwise with the following parameters: 50 10 0.1, and applied a minor allele frequency (MAF) filter of 1%. We inferred global ancestry proportions using ADMIXTURE (Alexander et al., 2009) on supervised mode for four ancestry clusters for each population merged with the references. After ADMIXTURE runs, we calculated the average proportion of European ancestry for each population. We used R to estimate the Spearman correlation (ρ) between the average proportion of European ancestry and allelic and genotypic frequencies for all populations.

To infer the ancestry of the genomic region flanking the *–13910C* > *T* SNP, we analyzed the complete chromosome 2, which includes the *MCM6* gene, for each dataset (LARGE-PD Project dataset and the merged dataset) without removing linked variants. First, for each dataset, all chromosomes were phased with shapeit4 (Delaneau et al., 2019) using the GRCh38 genetic map and the MCMC parameters: –mcmc-iterations 10b,1p,1b,1p,1b,1p,1b,1p,10m, which perform 10 burn-in iterations, followed by four paired runs of pruning and burn-in, and, finally, 10 main iterations of sampling. Then, we ran RFMix ver2 in order to infer the local ancestry (Maples et al., 2013) using the phased dataset with two expectation–maximization runs. After RFMix completion, we used the RFMix msp output, which includes the most likely assignment of ancestry per conditional random field point to obtain the size of the haplotypes per ancestry. The size was determined by considering the uninterrupted length (in base pairs) of the genomic region flanking the *–13910C* > *T* SNP until a switch in ancestry along the haplotype. We used in-house Perl and R scripts to determine the ancestry of the flanking region of *–13910^∗^T* allele and the length distribution of European haplotypes of the flanking region of the *MCM6* gene.^1^

### Natural Selection Analysis

In order to identify evidence of natural selection acting over the *MCM6* region, we applied two approaches. First, to identify a potential overrepresentation of European haplotypes due to recent, post-admixture natural selection acting over the *MCM6* region, we compared the average global European ancestry with the proportion of European haplotypes inferred by RFMix for each population. Moreover, we analyzed the length distribution of uninterrupted European haplotypes around the *–13910C* > *T* SNP to explore the genomic region dynamics. Second, we inferred the pattern of extended haplotype homozygosity (EHH) and the length distribution of derived haplotypes around the *–13910C* > *T* SNP in each population in order to identify whether the selection signal observed in the populations of European source is also observed in the admixed Pan-American populations. For this purpose, we used the rehh package (Gautier et al., 2017). Then, we estimated the integrated haplotype score (iHS), which indicates if a locus is under recent positive selection. This score compares the levels of linkage disequilibrium surrounding a positively selected allele with the ancestral allele background at the same position (Voight et al., 2006). For such iHS inference, we used the selscan software (Szpiech and Hernandez, 2014) with default parameters for phased information for each population. Finally, we calculated the genome-wide iHS *Z*-scores value by a normalization using the norm package, provided with selscan, in derived allele frequency bins with the –bin option set as 20. We calculated a two-tailed *p-*value of the SNPs based on the normalized iHS *Z*-scores by dividing the proportional rank of the statistic by the total number of values in the distribution.

## Results

### The *–13910^∗^T* Allele Distribution Is Correlated With European Ancestry in the Americas

**FIGURE 1:**
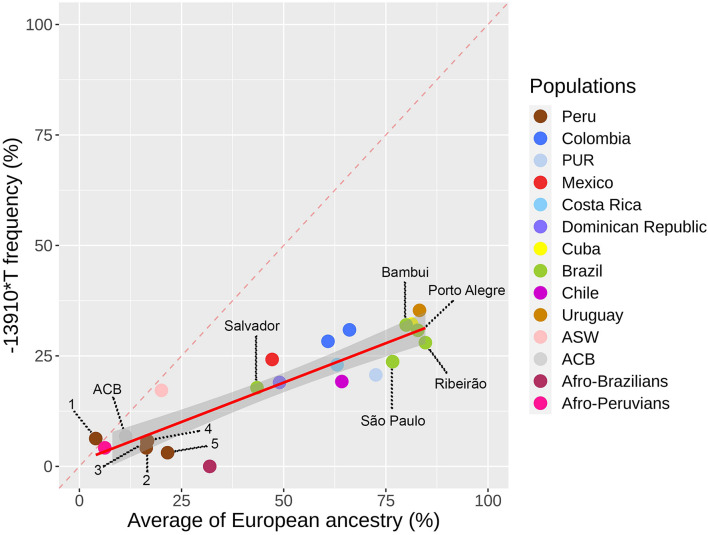
The –13910*T allele frequency for lactase persistence versus average of European ancestry in 12 Latin American countries. Colombia includes CLM and Bogota individuals. The red line between the samples represents a linear regression for the Pan-American populations. Peruvian populations: (1) Puno, (2) Trujillo, (3) Cusco, (4) Iquitos, and (5) Lima.

We explored the geographical distribution of the *–13910^∗^T* allele in 25 Pan-American populations of 12 countries. The highest frequency of the *–13910^∗^T* allele in the Americas occurs in the Uruguayan population (35%, Figure 1 and Supplementary Table 5). Conversely, the lowest frequencies were observed for Peruvian and African descendant populations. A positive correlation was observed between *–13910^∗^T* allele frequencies and the average proportion of European ancestry (ρ = 0.843, *p* = 4.414e-7). Consequently, we did not observe an overrepresentation of the *–13910^∗^T* allele compared with the average proportion of European ancestry in any population (Figure 1). Moreover, in Afro-Brazilians from Curitiba, we identified the *–14011^∗^T* (*–14011C > T*, rs145946881) and *–13915^∗^G* (*–13915T > G*, rs41380347) LP-associated alleles in low frequencies (0.2%).

**FIGURE 2:**
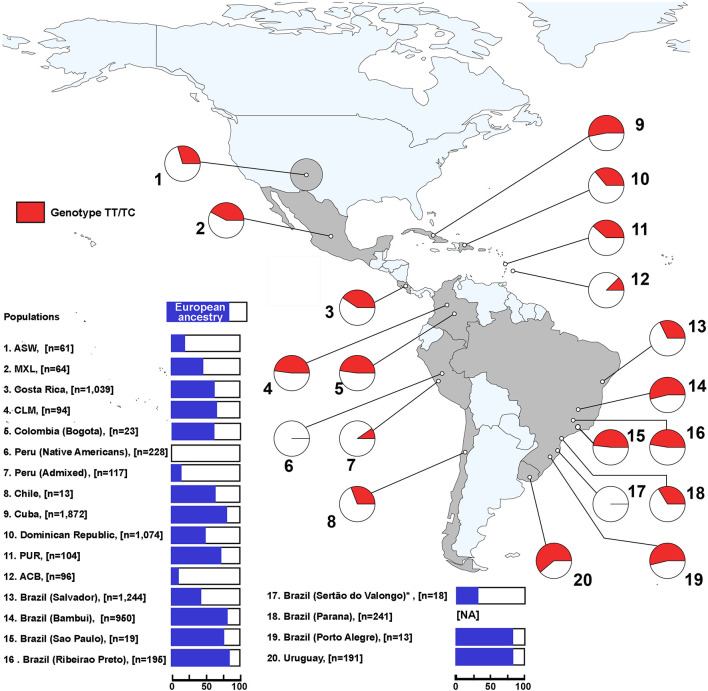
Geographical distribution of *TT* and *TC* genotypes of *–13910C* > *T* along the Americas. The red color in the pie charts represents the combined frequencies of *TT* and *TC* genotypes in each population. On the left, the blue color in the bar plots indicates the average proportion of European ancestry for each population. NA, no available data; ASW, African Americans from Southwest United States; MXL, Mexican ancestry from Los Angeles, United States; ACB, African Caribbeans from Barbados; PUR, Puerto Ricans from Puerto Rico; CLM, Colombians from Medellin. *European ancestry proportion for Afro-Brazilians from Sertão do Valongo was estimated using different samples from the same population from Luizon (2007).

Considering that the LP phenotype is a dominant trait, we explored the geographical distribution of *–13910^∗^T* allele carriers (*TT* and *TC* genotypes) as a proxy for the proportion of individuals with this phenotype. The observed genotype frequencies did not differ from those expected under Hardy–Weinberg equilibrium in all Pan-American admixed populations (Supplementary Table 5). Higher proportions of *T* allele carriers were observed in Uruguay (61%), Southeast–South Brazil (∼54%, including Bambui and Porto Alegre), and Cuba (53%), which also have higher proportions of European ancestry (Figure 2 and Supplementary Table 5), suggesting that more than half of the individuals of these samples are LP. Conversely, this inferred phenotype is absent in ancient and some present-day Peruvian Native Americans (Figure 2 and Supplementary Table 5). Furthermore, admixed Peruvians and African descendants (ASW, ACB, Afro-Peruvians, and Afro-Brazilians) have lower LP frequencies. Especially, Afro-Brazilians from Sertão do Valongo did not carry the *–13910^∗^T* allele nor other LP-associated alleles.

### The Haplotype Distribution of the *MCM6* Gene Reflects the History Along the Americas

**FIGURE 3:**
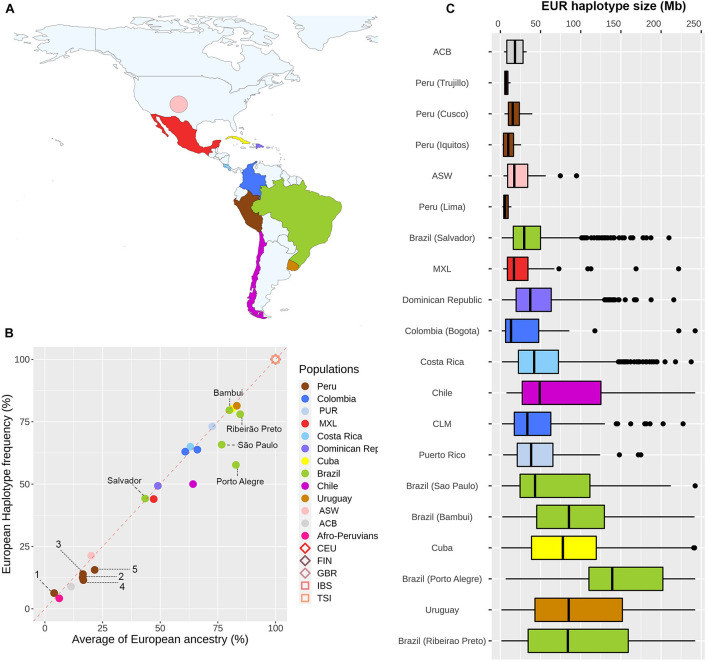
Distribution of counts and lengths of haplotypes of European ancestry in the vicinity of the *MCM6* gene along the Americas. **(A)** Colors indicate the countries included in the analyses. **(B)** European haplotype frequency vs. the average of European ancestry for each population (Uruguay and Bambui dots are overlapping the Cuba dot). European data (CEU, FIN, GBR, IBS, TSI) belong to the 1000 Genomes Project (1000 Genomes Project Consortium, Auton et al., 2015). Colombia includes CLM and Bogota individuals. Diamonds and squares refer to Northern and Southern European populations, respectively (European data are overlapped). Peruvian populations: (1) Puno, (2) Trujillo, (3) Cusco, (4) Iquitos, and (5) Lima. **(C)** Length distribution of European haplotypes that include the *MCM6* gene in Pan-American admixed populations. Populations with less than five European haplotypes were excluded.

To infer which forces are playing a significant role in the evolution of the *MCM6* gene in the Americas, we explored the distribution of counts and lengths of European haplotypes that include this gene. To address this goal, we performed a local ancestry inference for this locus and confirmed that the *–13910^∗^T* allele is observed only in European haplotypes in all Pan-American admixed populations. To determine if there is an overrepresentation of European haplotypes (with or without the *–13910^∗^T* allele) in the *MCM6* region, we estimated the proportion of uninterrupted European haplotypes and compared it to the genome-wide proportion of European ancestry for each population. Our results (Figure 3B) revealed a high correlation between European haplotype frequencies and the percentage of genome-wide European ancestry (ρ = 0.9435, *p* = 4.042e-6). This pattern suggests the possibility of neutral evolution of this genomic region after the admixture processes that gave rise to these Pan-American admixed populations.

We explored the admixture dynamics of this region by analyzing the length distribution of uninterrupted European haplotypes that include the *MCM6* gene. We observed a high density of small European segments in populations with a lower proportion of European ancestry (Figure 3C), such as Peruvians and African descendants, suggesting an early gene flow from Europeans. Furthermore, populations with higher European ancestry have a wide length distribution of European segments, indicating a continuous or multidate gene flow from Europeans. Both patterns are in agreement with historical records and population genetics studies.

**FIGURE 4:**
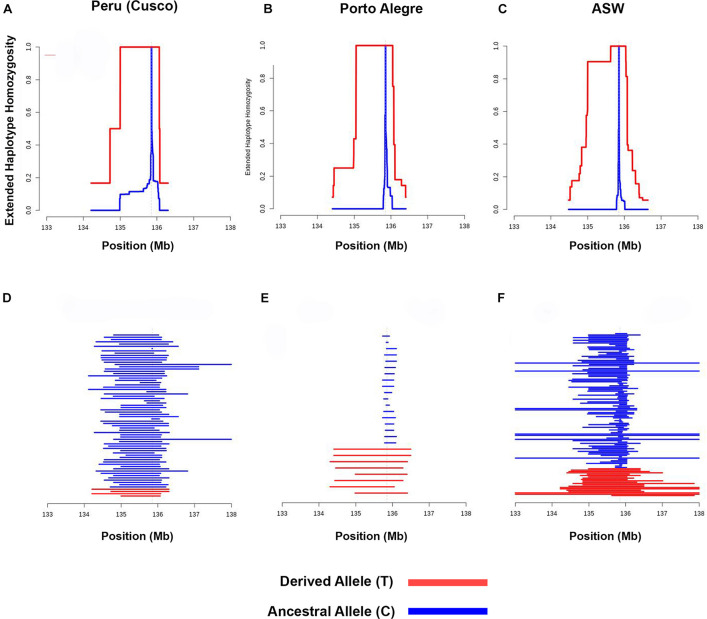
Extended haplotype homozygosity analyses. Extended haplotype homozygosity with –13910*T as the core allele in **(A)** Peruvians from Cusco, **(B)** Porto Alegre, and **(C)** ASW. Haplotype lengths for the derived and ancestral haplotypes with –13910*T as the core allele in **(D)** Peruvians from Cusco, **(E)** Porto Alegre, and **(F)** ASW. The derived allele (*T*) was observed only in European haplotypes, whereas the ancestral allele (*C*) was observed in European, African, and Native American haplotypes.

**FIGURE 5:**
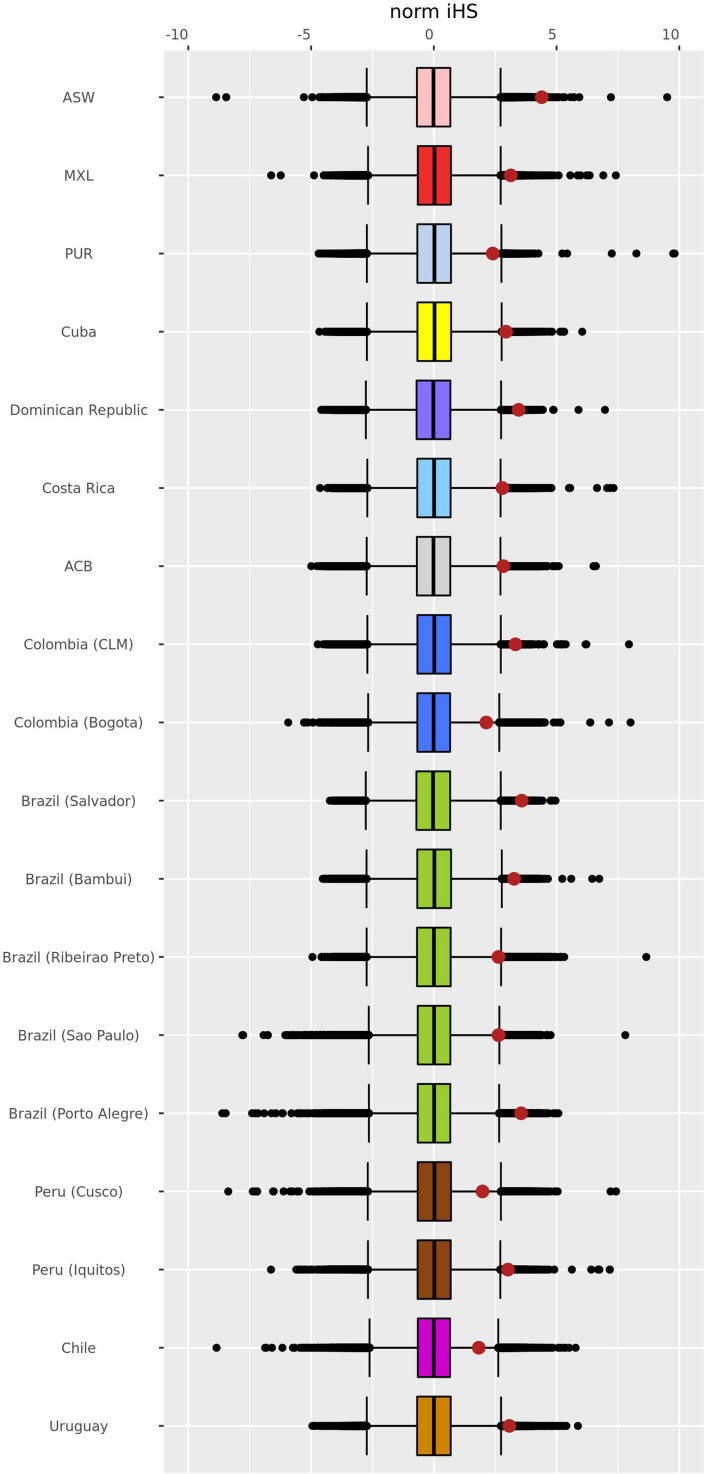
Genome-wide distribution of normalized iHS *Z*-scores. The scores were estimated using the genome-wide distribution of the unstandardized iHS in Pan-American admixed populations with –13910*T allelic frequencies > 5% and more than 10 individuals. After normalization, the mean and median are 0, and the standard deviation is 1. The whisker ends of the box plot indicate a value of ± 2.69, delimiting 99.3% of the distribution. The middle line inside the box plot corresponds to the median of the normalized iHS *Z*-score distribution. The red dots refer to normalized iHS *Z*-scores for the –13910*T allele. ASW, African Americans from Southwest United States; MXL, Mexican ancestry from Los Angeles, United States; PUR, Puerto Ricans from Puerto Rico; ACB, African Caribbeans from Barbados; CLM, Colombians from Medellin. Colors represent the countries just as the map in Figure 3A. Normalized iHS *Z*-scores and empirical *p-*values for the estimated –13910*T allele are described in Supplementary Table 5.

We performed EHH analysis for each population to evaluate the haplotypes and to compare their backgrounds for the *–13910^∗^T* allele with the ancestral *–13910^∗^C* allele. Most of the assessed populations presented a decay pattern of haplotype homozygosity around the *–13910^∗^T* allele (Supplementary Figure 2). Peruvians from Cusco and the populations of Bogota, Chile, and Porto Alegre have a linkage disequilibrium (LD) block of 1 Mb with 100% EHH that includes the derived allele (Figure 4 and Supplementary Figure 2). Observing the length of the EHH blocks (Figure 4 and Supplementary Figure 3), it is remarkable that African American and Porto Alegre populations have more extended and more homogeneous haplotypes for the derived allele in opposition to the haplotypes containing the *–13910^∗^C* allele (Figure 4). In both populations, all derived haplotypes are longer than 1 Mb. Specifically, in African Americans, some haplotypes reach 5 Mb. Moreover, several Pan-American populations presented iHS values greater than 2 (Supplementary Table 5), whereas African Americans presented the highest value (iHS = 4.2, Figure 5).

Interestingly, when we calculated the derived haplotype frequency, which includes the *–13910^∗^T* allele, in European haplotypes for each Pan-American population, the African Americans from Southwest United States (ASW) had the highest value (80%) (Supplementary Table 5). This frequency is quite similar to the frequency of the derived haplotype in Northern European populations (Supplementary Figure 4). Moreover, we observed that populations that were Spaniard or Portuguese colonies (Peru, Colombia, Mexico, Chile, Uruguay, and Brazil) had derived haplotype frequencies more similar to those of Iberians from the 1000 Genomes Project (Supplementary Figure 4). All these observations suggest that in Pan-American admixed populations, for the genomic segment that includes the *MCM6* gene, demography plays a more significant role than natural selection after the admixture processes.

## Discussion

LP is among the most strongly selected phenotypes in human populations; for LP, positive selection occurred during the last 5,000–10,000 years (Bersaglieri et al., 2004). In the Americas, there is no evidence of dairy consumption by native groups until the arrival of Europeans (Gade, 1999). Moreover, the digestion test performed in Native American people evidenced the highest incidence (>80%) of lactose intolerance in the Americas (Duncan and Scott, 1972; Caskey et al., 1977). This is consistent with the absence of the *–13910^∗^T* allele in ancient and present-day unadmixed Native Americans and with the hypothesis that this allele was probably introduced in the Americas by Europeans since the Colonial period. Furthermore, the identification of the *–14011^∗^T* and *–13915^∗^G* alleles in low frequencies in Afro-Brazilians from Curitiba agrees with the frequencies previously reported (Friedrich et al., 2012b; Paz-Y-Miño et al., 2016), corroborating the suggestion that the phenotypic variation observed in this study is mainly due to the *–13910C > T* variation of European origin.

In the present study, we conducted the most geographically extensive analysis of the *–13910^∗^T* allele spanning several Pan-American admixed populations (North American African Descendants and Latin Americans), demonstrating high positive correlation between the distribution of the derived allele and European ancestries, with no evidence of post-admixture positive selection. Furthermore, in light of the current Latin American nourishment policies, our results represent an important aspect to be considered for the understanding of the relationship between the consumption of dairy products and well-being across the continent.

We can address two limitations of our study. The first was the uneven representation of the population sampled within the countries: sample sizes varied from 13 individuals (Chile) to more than 1,000 individuals (Costa Rica, *n* = 1,039). However, if we restrict our analyses to countries with more than 30 sampled individuals, one may still observe the patterns described. Second, to maintain a balance between individuals and SNP density, we used two separate datasets for the analyses (LARGE-PD and a merged dataset). However, as the study was focused on the *–13910C* > *T* SNP and its flanking haplotypes of European origin, the genomic ancestry would still be the same if inferred with a different set of SNPs, as long as the inference is performed with the same SNP density.

### Impact of European Colonization in the Distribution of LP Alleles

European immigration had varied scales and influences across Central and South American regions. Despite occurring since the fifteenth century, European migration to the Americas was intensified during the late nineteenth century. Between the 1870s and the 1930s, approximately 13 million Europeans arrived in Latin America, of which more than 90% migrated to Argentina, Brazil, Cuba, and Uruguay (Sánchez-Alonso, 2019). In agreement, higher proportions of European ancestry have been observed in Brazilian populations (except for Salvador), Cubans, and Uruguayans. Also, the length distribution of European haplotypes is wider in these populations, which is consistent with demographic data of a more continuous European gene flow.

Previous studies and historical records reported earlier European colonization in Northeast Brazil and higher proportions of African ancestry in this region (Instituto Brasileiro de Geografia e Estatística, 2000; Pena et al., 2011; Kehdy et al., 2015). We observed in the Salvador sample (Northeast Brazil) a low percentage of European ancestry correlated with a low *–13910^∗^T* allele frequency and a low European haplotype frequency. Moreover, a short European haplotype was observed, possibly introduced earlier in the Salvador population than in other Brazilian populations. A similar pattern was observed in African descendant populations, in which the lowest average of European ancestry was detected. In African Americans from Southwest United States, the European ancestry corresponds to a minor proportion, which explains the low frequency of the *–13910^∗^T* allele in this population. Interestingly, the largest part of the European immigrants who came to the United States originated from countries where the frequencies of the *–13910^∗^T* allele are typically high (Bersaglieri et al., 2004; Smith et al., 2009; Itan et al., 2010), resulting in the presence of this variant in most of the European haplotypes observed in African Americans. The patterns observed in our work are consistent with one-way admixture events that have been inferred for these populations in previous studies (Harris et al., 2018; Ongaro et al., 2019; Gouveia et al., 2020).

In the admixed Peruvian populations, we also observed a low *–13910^∗^T* allele frequency, a low European haplotype frequency, and a short European haplotype – similarly to those observed in African descendant populations, including Afro-Peruvians. This pattern is concordant with the hypothesis that the admixture between European colonizers (mainly Spanish) and Native Americans occurred mostly after the independence of Peru in 1824, about 300 years after the European settlement, resulting in the low European ancestry reported in Peruvian and Afro-Peruvian populations, which range between 14 and 22% (Harris et al., 2018).

Genetic studies indicated that the admixture between the Caribbeans and Europeans occurred shortly after the arrival of these populations in the American continent (Moreno-Estrada et al., 2013), resulting in the short European haplotypes observed in populations from Costa Rica, Dominican Republic, Barbados, and Puerto Rico. In contrast to these Central American countries, the wider European haplotype observed in Cubans was possibly due to the marked European migration to this country in the early twentieth century (Salzano and Bortolini, 2005).

Altogether, our results show that the distribution of lengths and frequencies related to the *–13910^∗^T* allele reflects the demographic history of Pan-American admixed populations.

### LP in an Afro-Brazilian Quilombo Community

Between the sixteenth and mid-nineteenth centuries, Europeans brought to Brazil around 4 million enslaved Africans (Instituto Brasileiro de Geografia e Estatística, 2000). Africans and their descendants formed communities named Quilombos, a form of resistance to slavery and a stronghold for African culture (Moura, 2001; Raggio et al., 2018). The quilombola community of Sertão do Valongo (Santa Catarina State, Southern Brazil) has a reported African genomic ancestry of 68.1% and a European one of 31.9%, therefore with no reported Native American ancestry (Luizon, 2007). The absence of the *–13910^∗^T* allele in Afro-Brazilians from Sertão do Valongo and, consequently, the absence of the inferred LP phenotype agree with the settlement history of the community marked by the small and early European contribution. Quilombola communities, in general, are not genetically isolated from other populations, although the degree of interaction with neighboring groups may vary (de Almeida, 2010). Hence, as each community has a distinct population structure, settlement history, and cultural practices, the evaluation of other quilombola communities is essential to the understanding of the *–13910^∗^T* allele distribution in these groups.

### Lack of Postadmixture Selective Pressure for LP in the Pan-American Admixed Populations

The high correlation between European haplotype proportion and European genome-wide proportion suggests no selective pressure over the *MCM6* gene after admixture. Nonetheless, our EHH analysis showed that several populations have longer LD blocks containing the derived allele. Moreover, the iHS values for these populations were greater than 2, which indicates some level of positive selection (Voight et al., 2006). Considering that there is no overrepresentation of European haplotypes in the *MCM6* gene and that the length distribution of these haplotypes reflects demographic history, we hypothesized that the detected iHS signals result from positive selection in the source population. Specifically, African Americans, which had the highest iHS value, have Northern Europe as the main source of their admixture (Ongaro et al., 2019; Gouveia et al., 2020). Other populations with higher European ancestry levels (i.e., Brazilian populations) could have moderate iHS values due to the highly diverse European sources introduced into Brazil during the last century.

In contrast, by evaluating goat herders from Coquimbo (Chile), Montalva et al. (2019) reported a significant enrichment for European ancestry in the *LCT* gene when compared to genome-wide European ancestry, suggesting recent positive selection after admixture. However, the enrichment for European ancestry in the *LCT* gene was absent in urban non-pastoralist Latin American populations evaluated [e.g., MXL, CLM, and PEL (Peruvians in Lima, Peru) from the 1000 Genomes Project]. Thus, the lack of post-admixture selective pressure observed in our study – which datasets included MXL, CLM (1000 Genomes Project Consortium, Auton et al., 2015), and Peruvians (Harris et al., 2018; Borda et al., 2020) – agrees with Montalva et al. (2019), supporting their hypothesis of specific adaptation to milking agropastoralism in the Coquimbo population.

It should be noted that admixture processes could hinder the detection of selection signals due to changes in the allelic frequencies and linkage disequilibrium patterns (Lohmueller et al., 2011; Hamid et al., 2021). Furthermore, a recent study shows that a strong selective pressure of an adaptive phenotype could lead to genome-wide changes, modifying chromosome regions not directly involved in the phenotype, which can potentially bias the ancestry inference (Lohmueller et al., 2011; Hamid et al., 2021). Therefore, although it is unlikely that the LP phenotype has promoted differential survival in the American continent in the last centuries, we cannot ignore that the aspects early mentioned may have influenced our results.

### Impact of Our Results on Public Health Policies

Dairy plays an essential role in human nutrition because of its supply of protein, lipids, and micronutrients, such as calcium, magnesium, and vitamins B_5_ and B_12_, among others (Muehlhoff et al., 2013). However, it is necessary to emphasize that, in recent years, the consumption benefits of dairy in adulthood have been questioned. Whereas some studies have linked its consumption to the development of cardiovascular diseases and obesity – possibly because of the fat content of milk (Segall, 1994; Berkey et al., 2005; Muehlhoff et al., 2013) – other studies have suggested that milk and dairy intake could reduce the risk of metabolic syndrome, diabetes, and cancer development (Elwood et al., 2007, 2008).

Dairy consumption is frequently encouraged – and even financed – by governmental initiatives to fight famine in Latin American countries due to these products’ unarguable nutrient content. The “Programa de Abasto Social de Leche LICONSA” in Mexico (created in 1944), the “Programa Nacional de Alimentación Complementaria” or PNAC in Chile (created in 1952), the “Programa del Vaso de Leche” or PVL in Peru (created in 1984), and the “Programa de Aquisição de Alimentos” in Brazil (created in 2003) are examples of governmental initiatives (Uauy et al., 2001; Stifel and Alderman, 2006; Hespanhol, 2013; Morales-Ruán Mdel et al., 2013). Moreover, in Cuba, Costa Rica, México, Venezuela, Colombia, and Uruguay, dietary guidelines also recommend the consumption of dairy products (Instituto Nacional de Nutrición, 1990; Porrata-Maury, 2004; Comisión Intersectorial de Guías Alimentarias Para Costa Rica Ministerio de Salud, 2010; Secretaria de Salud de México, 2010; Instituto Colombiano de Bienestar Familiar and FAO Colombia, 2015; Dirección General de la Salud Ministerio de Salud de Uruguay, 2019). These initiatives follow the US program pattern, in which the dietary guidelines include milk as an essential nourishment element since its first publication (Bertron et al., 1999; Jacobs et al., 2020). However, the US programs relied on early nutrition scientific studies developed mostly in Northern European populations (Jacobs et al., 2020), where a high prevalence of LP is observed (Ingram et al., 2009). Considering the prevalence of the inferred LNP phenotype in the Americas and the complex ancestry of Pan-American populations, dairy intake could contribute to the generation of health issues due to the development of lactose intolerance–associated events.

It is important to highlight that the gut microbiota composition and epigenetics modifications affect the manifestation of lactose intolerance–associated events, attenuating or eliminating them in individuals who typically cannot digest the disaccharide (Zhong et al., 2004; He et al., 2008; Labrie et al., 2016; Anguita-Ruiz et al., 2020). The manifestation of these events is also dependent on the lactose concentration of the food, which tends to be reduced in fermented dairy foods, such as cheese and yogurt (Buttriss, 1997). According to this, cultural and colonic adaptation mechanisms could explain the consumption of milk and dairy products without causing gastrointestinal events in non-carriers of genetic variants associated with LP (Hertzler and Savaiano, 1996; Segurel et al., 2020; Bleasdale et al., 2021). Thus, the absence of the *–13910^∗^T* allele alone may not be completely predictive of lactose intolerance, especially considering the existence of other alleles of non-European origin associated with the LP phenotype (Tishkoff et al., 2007; Ranciaro et al., 2014), although the low frequencies of such alleles in Pan-American admixed populations (Friedrich et al., 2012b; Paz-Y-Miño et al., 2016) reinforce the effectiveness of the *–13910^∗^T* allele for the genetic diagnosis of LP.

Our results revealed that, in the Americas, only a few populations from Cuba, Brazil (Bambui and Porto Alegre), and Uruguay have an elevated proportion (greater than 50%) of individuals who are likely to be lactase persistent, agreeing with the fact that these countries (except for Cuba) are among the largest consumers of dairy products (excluding butter) per year in the continent: 175.3 and 141.8 kg per capita for Uruguayans and Brazilians, respectively (Food and Agriculture Organization of the United Nations, 2018). A lower consumption in other Latin American countries (Food and Agriculture Organization of the United Nations, 2018) is associated with the prevalence of the LNP phenotype, which is consistent with the recent introduction of cattle and, consequently, with the consumption of dairy products in the continent (Gade, 1999). Therefore, traditional consumption of these products by Native American populations is rare (Kiple and Ornelas, 2001). However, it should be noted that European immigrants (and their descendants) have established such practices in culinary in America, resulting in the recent development of typically milk-based dishes in several countries of this continent (Albala, 2011; Wiley, 2015). The socioeconomic status of Latin American countries also contributes to such mentioned lower levels, as consumption of dairy products is known to be lower in developing countries than in developed ones, despite the changes over the last decades due to increasing dairy consumption (Bermudez and Tucker, 2003; Muehlhoff et al., 2013).

The reduced frequency of the *–13910^∗^T* allele may be a hint that dairy products are not the best option for dietary guidelines in populations with lower proportions of European ancestry, such as Peruvian and African descendant populations (African Americans, African Caribbeans, Afro-Brazilians from Curitiba, and the quilombola community of Sertão do Valongo). According to the data discussion above, European-biased policies that include dairy products should be rediscussed and reconsidered. Furthermore, until the role of dairy intake in the pathogenesis of complex diseases is fully understood, alternative milk products and plant-based dairy substitutes would possibly be a better option for the dietary guidelines of America.

## Data Availability Statement

Newly generated sequences were deposited in the GenBank at NCBI website under the accession numbers MZ362598–MZ362856.

## Ethics Statement

The studies involving human participants were reviewed and approved by Brazilian CONEP (Comissão Nacional de Ética em Pesquisa). The patients/participants provided their written informed consent to participate in this study.

## Author Contributions

MHB, CS, and ViB designed the study. AG and NS performed the Sanger sequencing and the analysis of Afro-Brazilian populations. MHB, MP-E, RL, and IR contributed with samples and reagents. CS, MD, J-YM, RK, KN, SW, MLB, ML-C, HG, OC, CP, ET-S, IM, ED, VR, AL, VT, VaB, HF, CR, AS-S, BS-L, PC-C, WF, GA, HA, and CA-B generated the datasets and were responsible by them. ViB, MHB, AG, NS, GA, MMe, TL, MS, CS, DL, MMa, and TO’C, analyzed the data. AG, NS, MHB, and ViB drafted the manuscript. GAC, MMe, TL, MP-E, TO’C, OC, MD, and ET-S critically edited the manuscript. All authors contributed to the article and approved the submitted version.

## Conflict of Interest

The authors declare that the research was conducted in the absence of any commercial or financial relationships that could be construed as a potential conflict of interest.

## Publisher’s Note

All claims expressed in this article are solely those of the authors and do not necessarily represent those of their affiliated organizations, or those of the publisher, the editors and the reviewers. Any product that may be evaluated in this article, or claim that may be made by its manufacturer, is not guaranteed or endorsed by the publisher.

## Footnotes

1 All Perl and R scripts used for this study are freely available at: https://github.com/vicbp1/Lactase-persistance-in-the-Americas.git.
