# Supplementary material for: Tracing the Distribution of European Lactase Persistence Genotypes Along the Americas
Source: Front Genet. 2021 Sep 22;12:671079. doi: 10.3389/fgene.2021.671079 (PMC8493957; doi:10.3389/fgene.2021.671079)
Supplement: Supplementary file 1 [file Data_Sheet_1.docx]

**Supplementary Material**

**Tracing the distribution of European lactase persistence genotypes along the Americas**

Ana Cecília Guimarães Alves ^1,3^, Natalie Mary Sukow ^1^, Gabriel Adelman Cipolla ^1^, Marla Mendes ^2^, Thiago P. Leal ^2^, Maria Luiza Petzl-Erler ^1,3^, Ricardo Lehtonen Rodrigues de Souza ^3,4^, Ilíada Rainha de Souza ^1,5^, Cesar Sanchez ^6^, Meddly Santolalla ^7^, Douglas Loesch ^8^, Michael Dean ^9^, Moara Machado ^2^, Jee-Young Moon ^10^, Robert Kaplan ^10,11^, Kari E. North ^12^, Scott Weiss ^13^, Mauricio L. Barreto ^14,15^, M. Fernanda Lima-Costa ^16,17^, Heinner Guio ^6,18^, Omar Cáceres ^6,19^, Carlos Padilla ^6^, Eduardo Tarazona-Santos ^2^, Ignacio F. Mata ^20,21,22^, Elena Dieguez ^23^, Víctor Raggio ^24^, Andres Lescano ^23^, Vitor Tumas ^25^, Vanderci Borges ^26^, Henrique B. Ferraz ^26^, Carlos R. Rieder ^27^, Artur Schumacher-Schuh ^28,29^, Bruno L. Santos-Lobato ^30^, Pedro Chana-Cuevas ^31^, William Fernandez ^32^, Gonzalo Arboleda ^32^, Humberto Arboleda ^32^, Carlos E. Arboleda-Bustos ^32^, Timothy D. O'Connor ^8,33,34^, Marcia Holsbach Beltrame ^1,3†^, and Victor Borda ^8†^

^†^These authors have contributed equally to this work and share last authorship.

# **Supplementary Figures**


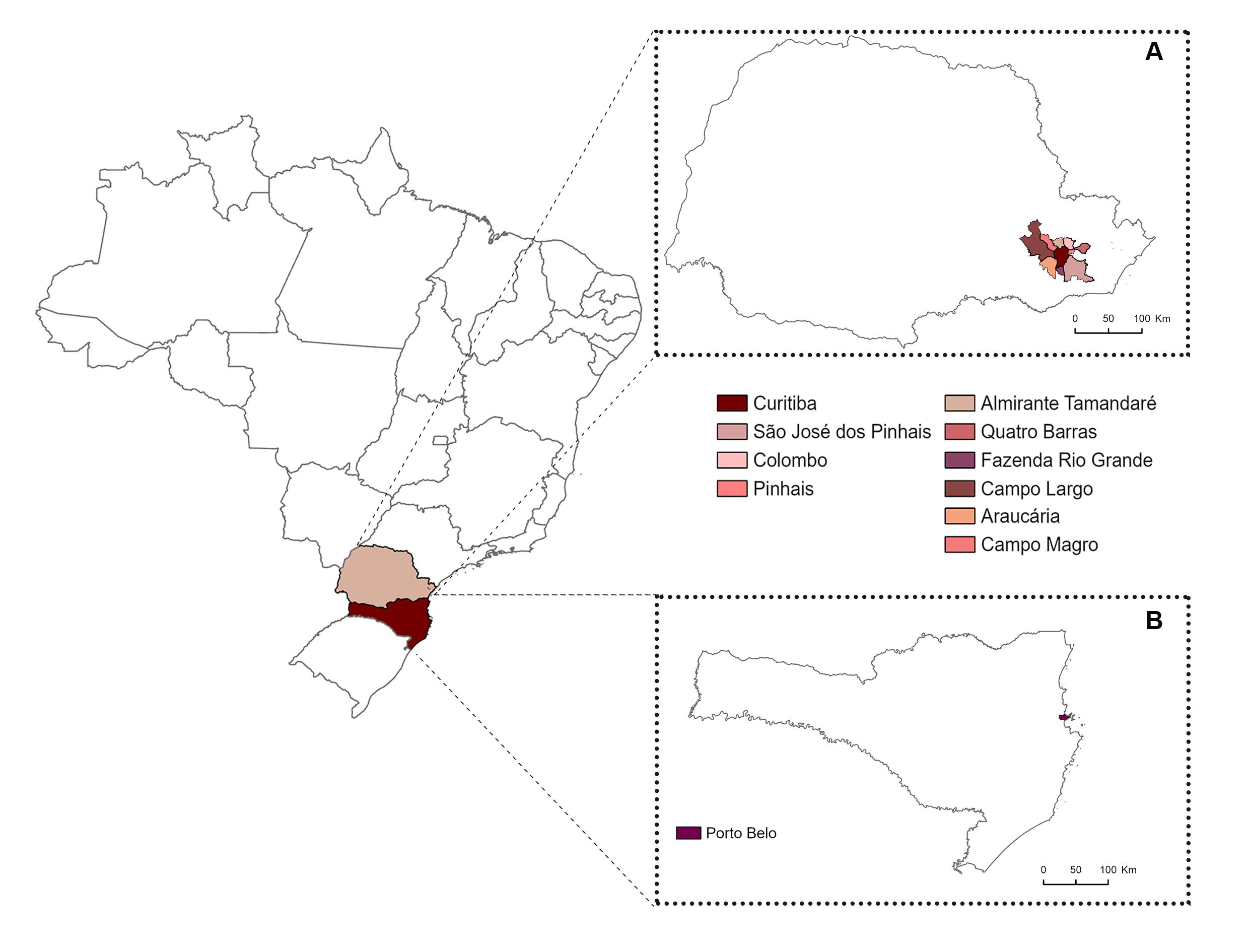


**Supplementary Figure 1. Location of sampled Afro-Brazilians for the Sanger sequencing of intron 13 from *MCM6* gene.** Individuals were sampled from A) Curitiba (Paraná state) and its metropolitan region and B) Sertão do Valongo (Santa Catarina state).


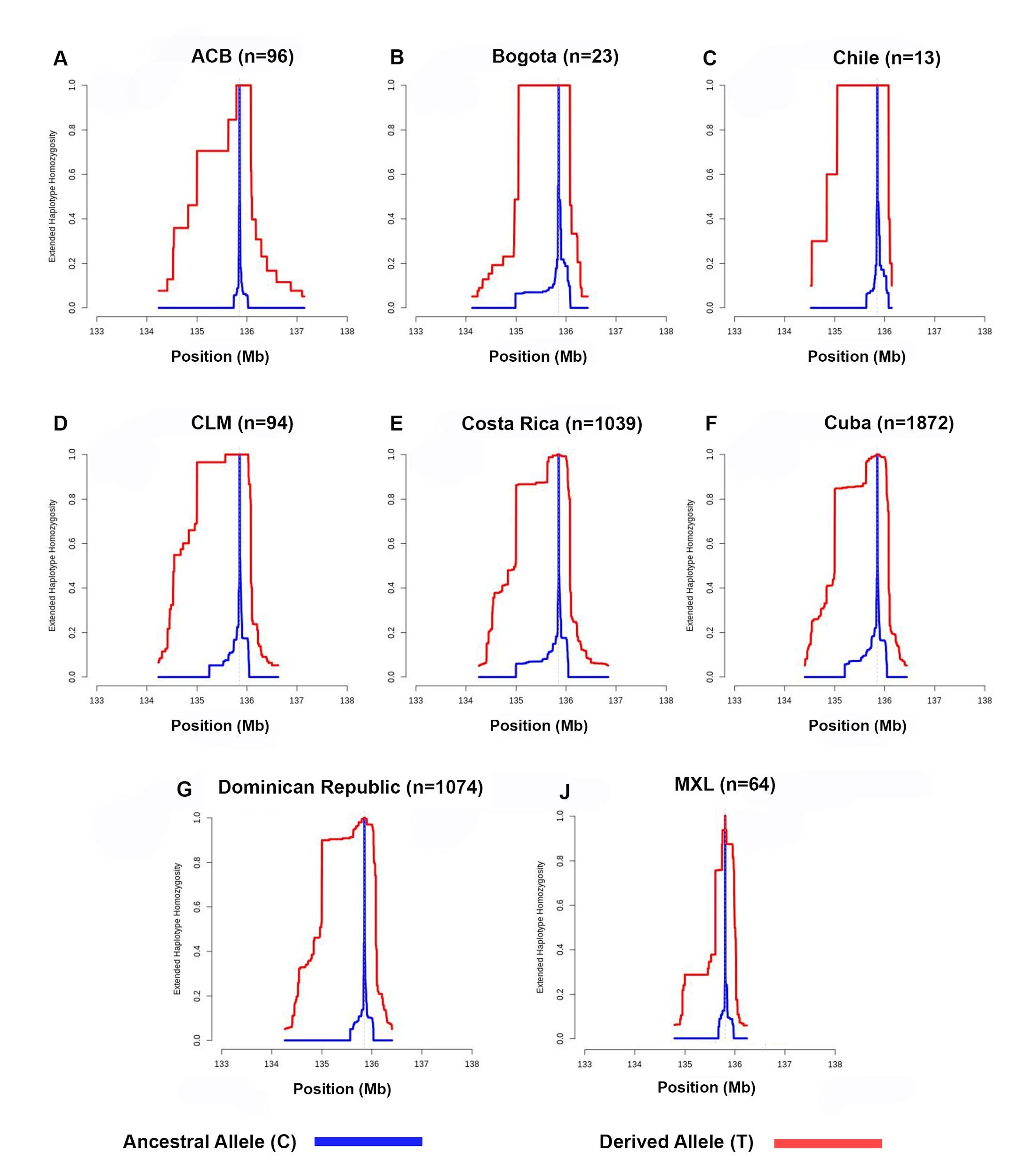


**Supplementary Figure 2. Pattern of Extended Haplotype Homozygosity in Pan-American populations.** The core allele corresponds to the *–13910*T* allele in the *MCM6* gene.


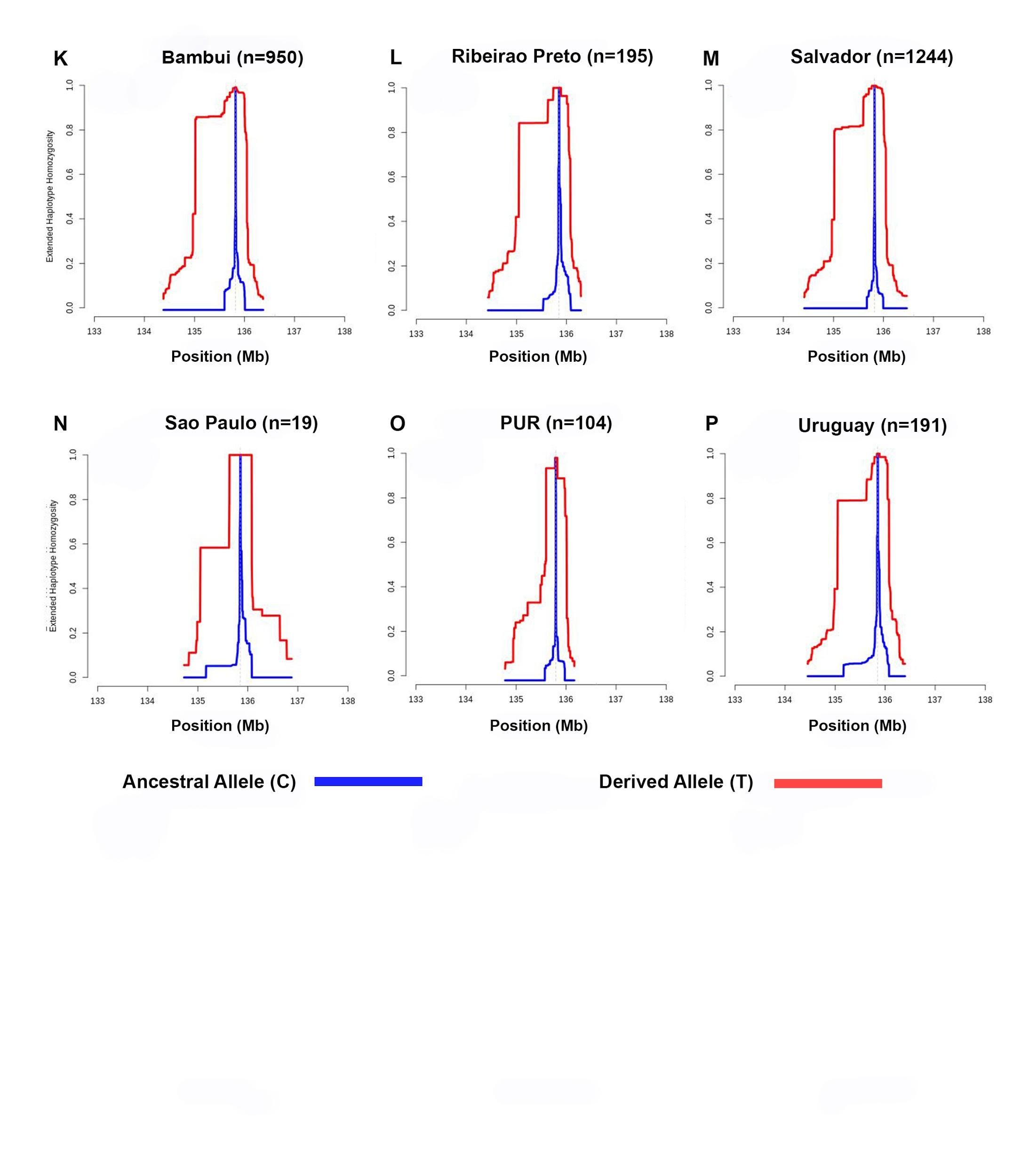


**Supplementary Figure 2 (Continuation). Pattern of Extended Haplotype Homozygosity in Pan-American populations.** The core allele corresponds to the *–13910*T* allele in the *MCM6* gene.


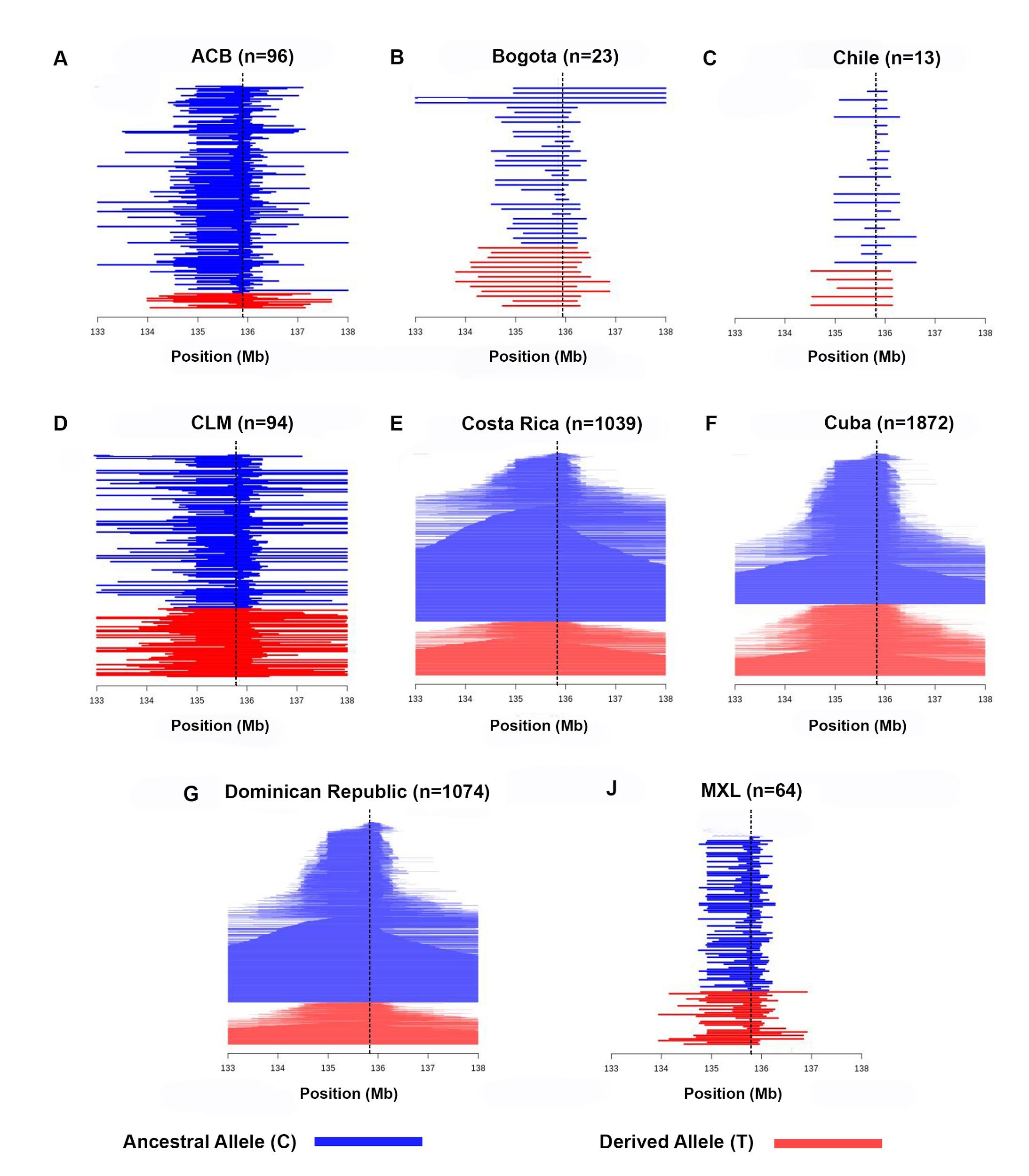


**Supplementary Figure 3. Lengths of Extended Haplotype Homozygosity in Pan-American populations.** The core allele corresponds to the *–13910*T* allele in the *MCM6* gene.


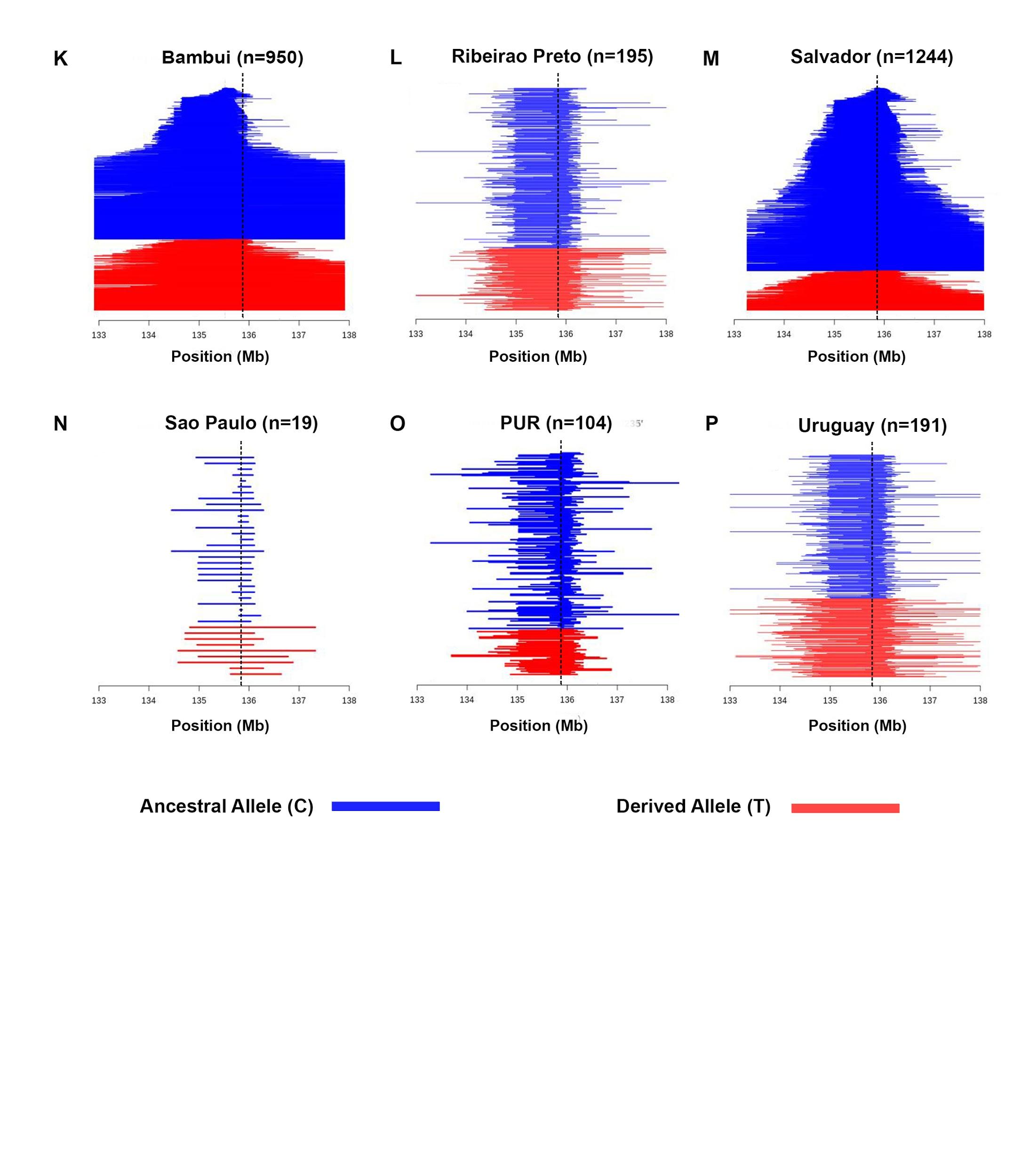


**Supplementary Figure 3 (Continuation). Lengths of Extended Haplotype Homozygosity in Pan-American populations.** The core allele corresponds to the *–13910*T* allele in the *MCM6* gene.


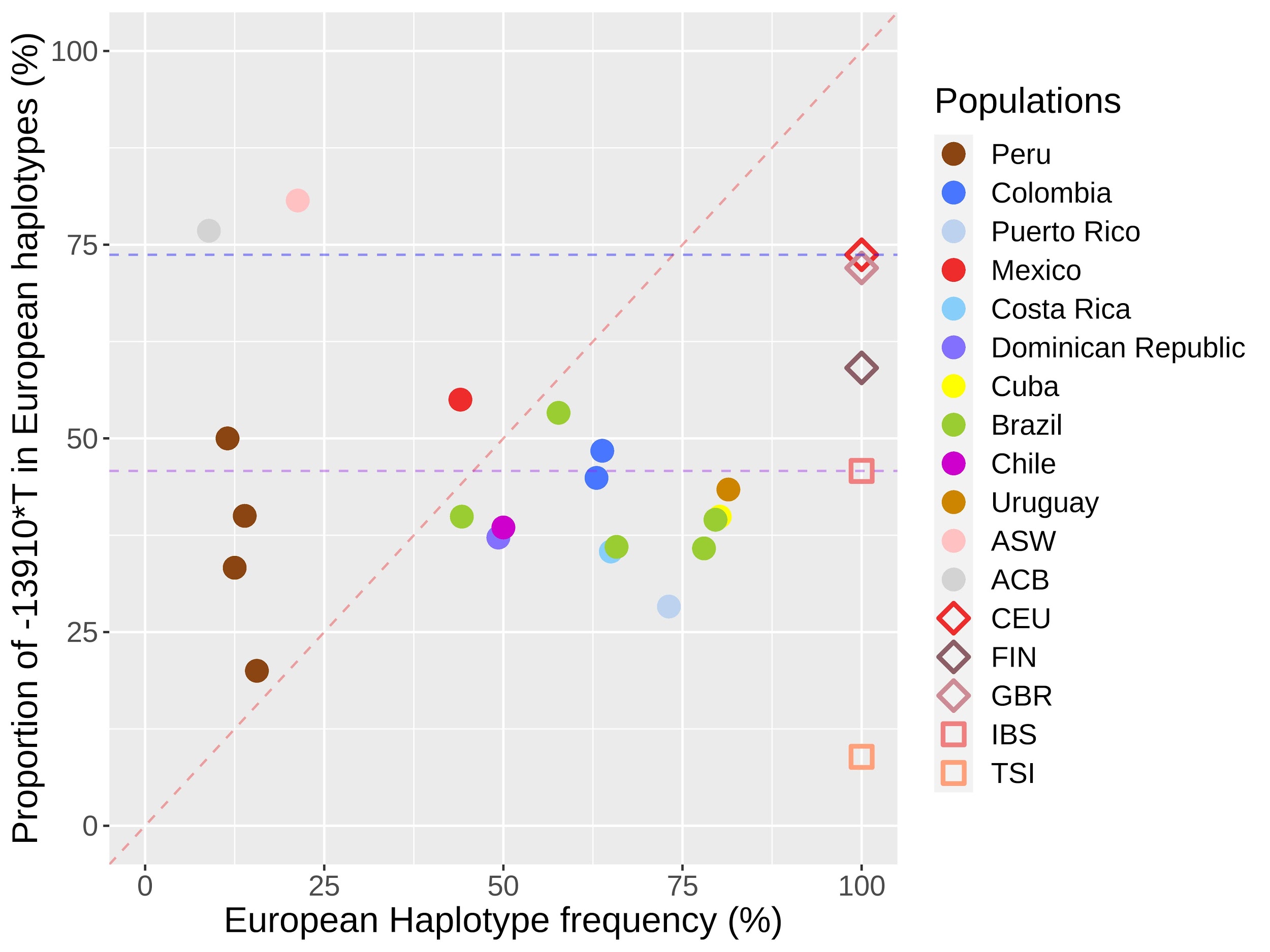


**Supplementary Figure 4. Proportion of the *–13910*T* allele in European haplotypes vs Proportion of European haplotypes in the *MCM6* gene in each population.** Blue and purple dashed lines correspond to the frequency of the derived allele in CEU (Northern European ancestry) and IBS (Southern European ancestry), respectively. Colombia includes CLM and Bogota individuals. Also, Diamonds and squares belong to Northern and Southern European populations, respectively.

#

#

# **Supplementary Tables captions**

**Supplementary Table 1.** Detailed information of the population included in this study.

**Supplementary Table 2.** Concentration and volume of reagents used per sample for the PCR reaction.

**Supplementary Table 3.** Protocol used for PCR reaction.

**Supplementary Table 4.** Afro-Brazilian individual genotype annotation for the *–13910C>T* SNP (rs4988235) and Genbank Accession codes for each individual.

**Supplementary Table 5.** Frequencies (allelic, genotypic and haplotypic), European ancestry and iHS values calculated for each population.
